# Exploring Response Inhibition, the Behavioral Inhibition System and Possible Sex Differences in Athletes and Non-Athletes

**DOI:** 10.3390/ijerph20146340

**Published:** 2023-07-10

**Authors:** Lina J. K. Eriksson, Örjan Sundin, Billy Jansson

**Affiliations:** 1 Department of Psychology and Social Work, Mid Sweden University, 83125 Östersund, Sweden; 2 Católica Research Centre for Psychological-Family and Social Wellbeing, Universidade Católica Portugesa, 1649-023 Lisboa, Portugal

**Keywords:** behavior mechanisms, executive function, goals, personality

## Abstract

Background: The objective of this study was to revisit the question concerning whether athletes are better than non-athletes at fundamental cognitive abilities, such as inhibitory control, in addition to also focusing on motivational dispositions and possible sex differences. Adding the latter could be crucial since both inhibitory control and motivational dispositions, such as approach and avoidance, are central to goal-directed behavior. Methods: This study’s sample was composed of 93 participants (40 males): 29 biathletes; 30 alpine skiers; and 34 non-athletes. A non-sport-specific stop-signal task was used for the assessment of inhibitory control in terms of response inhibition, and the motivational dispositions were assessed with the BIS/BAS scales. Results: The results showed that there were no differences between the two different sports or non-athletes with regard to response inhibition. However, females showed significantly slower response inhibition than males (*p* = 0.018) and scored significantly higher on the trait variable BIS (*p* < 0.001). Conclusions: The results from this study suggest that it might be meaningful to explore the contribution of sex differences and motivational dispositions on response inhibition in conjunction with different types of sports.

## 1. Introduction

The question of whether experience in sports is related to performance on basic tests of cognitive abilities is not new but still lacks a straight answer since previous findings are contradictory, e.g., [1,2,3,4]. Cognitive abilities can also be referred to as executive functions (EF), and they are essential for the coordination of behavior in line with an internal goal [5] and some kind of involvement of EFs in order to reach a goal. The striving toward a goal can also be explained in terms of motivational dispositions, such as approach and avoidance motivation. Most sports place demands on the EFs [6], and studies have demonstrated that EFs can predict success among top soccer players [7,8]. However, EFs and motivational dispositions are both central to goal-directed behavior, and it has previously been suggested that there is an interaction between cognitive control and motivation [5]. Consequently, approach and avoidance motivation should, therefore, also be considered when studying EFs in relation to sports. 

In the literature, three of the most frequently occurring EFs are (1) mental shifting/cognitive flexibility, (2) working memory, and (3) inhibition of prepotent (i.e., automatically processed) responses [9,10,11]. Inhibitory control is an EF that refers to the ability to suppress a prepotent response, impulse, or inappropriate thought [12]. Exercising inhibitory control enables humans to make a choice or to change [13], to choose how to behave or react, and helps control impulses [9]. Inhibitory control has been studied using a variety of experimental paradigms, one of these being the stop-signal paradigm [14,15], in which the inhibitory process can be measured behaviorally. The stop-signal task (SST) contains two tasks, a primary reaction task which requires a speeded response and a secondary stop task which requires inhibition of the response [16]. An SST enables the estimation of stop-signal reaction time (SSRT), and shorter SSRT indicates better inhibitory ability. It is important to recognize that SSRT is not an estimate of a single inhibitory function; rather, it captures a chain of processes that results in a response being withheld [17]. The evidence concerning whether sex differences exist in cognitive processes, such as response inhibition, is inconclusive [18]. However, Gaillard et al. [19] showed that males demonstrated significantly shorter SSRT than females in a sample of non-athletes. They found brain activation differences between males and females during SST performance, which could be one explanation for the differences in the behavioral outcome (i.e., SSRT). 

The stop-signal paradigm has previously been used to investigate inhibitory control in various sports. In soccer, talented male soccer players at young age outperformed amateur players [4] or sub-elite players on response inhibition [20]. Greater inhibitory control, in terms of response inhibition, has also been found in volleyball players compared to non-athletes [21], lower-level badminton players [22], and in male tennis players representing an open skill sport compared to male swimmers representing a closed skill sport and non-athletes [23]. However, Liao et al. [24] found no differences in response inhibition between elite badminton players and non-athletes. Hagyard et al. [25] argued that inhibitory control develops longitudinally and have further demonstrated that it relates to sport performance, which increases with greater sport expertise. However, Beavan et al. [1] showed that the number of years of experience in sports does not explain EF performance in football players. In high-level football players, changes in cognitive abilities seem to develop parallel to general populations. Krenn et al. [26] have suggested that for EFs, the type of sports may be more relevant than levels of expertise. Furthermore, in relation to sports, sex differences and/or motivational dispositions have not thoroughly been examined. 

A frequently used definition of sports includes dividing sports into three different types. The three types are (1) interceptive sports (e.g., tennis, boxing, biathlon, alpine skiing), (2) strategic sports (e.g., soccer, volleyball, hockey, basketball), and (3) static sports (e.g., swimming, long-distance running; see [27]). In interceptive sports, both gross and fine motor responses are included in the range of interceptive action, and these types of sports involve the coordination between the body and an object in the environment [28]. Hence, it seems that inhibitory control could be of importance for performance in interceptive sports, and the current study aimed to investigate response inhibition in athletes from two sports that can be viewed as interceptive, biathlon and alpine skiing, and non-athletes. Although biathlon and alpine skiing can both be defined as interceptive sports, it could be argued that precision shooting in biathlon may be defined as a static sport in that the sporting environment is relatively stable. Furthermore, precision shooting is a complex motor activity that requires a rapid execution [29], something that is not required to the same extent in alpine ski racing. Therefore, it is interesting to investigate if the differences in demands within these sports could be associated with differences in terms of response inhibition. 

As mentioned previously, motivation plays an important role in cognitive control, and motivational dispositions can be described in terms of approach and avoidance behaviors. One of the most frequently used theories to explain approach and avoidance motivation is the Reinforcement Sensitivity Theory [30,31]. The revised version of the Reinforcement Sensitivity Theory (rRST; [32,33]) describes three neuropsychological systems, and individual differences in the functioning of these neural systems are suggested to explain individual differences in behavior and emotion [34]. The three neural systems are the Behavioral Approach System (BAS), the Fight–Flight–Freeze System (FFFS), and the Behavioral Inhibition System (BIS; [33,35]). Individual differences in the BAS reflect sensitivity to reward, while the differences in the BIS and the FFFS reflect sensitivity to punishment. The BIS is sensitive to the so-called “goal conflict” [36]. In essence, a goal conflict has to do with conflicts between competing available goals. The most evident is the approach (i.e., BAS) and avoidance (i.e., FFFS) conflicts, but the BIS is also activated by FFFS-FFFS and BAS-BAS conflicts. The BIS can also be viewed as a personality factor that encompasses proneness to worry and anxious rumination [37]. In a large sample of non-athletes, Jorm et al. [38] have demonstrated that females scored higher levels of BIS than males, especially in the younger age groups. It has repeatedly been shown that adult females scored higher than males on BIS but not on BAS [39,40,41,42]. Moreover, the rRST has been used to investigate, for example, mental toughness [43,44], choking under pressure [45], and high-risk sports [46]. The SST has previously been used in combination with the BIS as measured by the rRST [47,48] and been suggested to impose a goal conflict since it presented two conflicting goals that required reaction to a primary task as well as an inhibition to stop-signals. In a sample of non-athlete adults, [49] higher levels of BIS were accompanied by a poorer ability to inhibit responses and associated with decreased accuracy in terms of correct responses. 

Previous findings on whether extensive experience in sports is related to performance on basic tests of cognitive abilities are contradictory, and few previous studies have given attention to motivational dispositions, such as the BIS and sex differences. The goal of the present research was, therefore, to investigate possible differences in response inhibition in non-athletes and athletes from two different sports, alpine skiing and biathlon. Response inhibition (indexed by SSRT) was assessed by a non-sport-specific stop-signal task. The hypothesis is that biathletes have shorter SSRT than alpine skiers and non-athletes due to the fact that precision shooting in biathlon can be defined as static and is a complex motor activity that requires rapid execution. Furthermore, the association between response inhibition and the BIS and sex differences in response inhibition and the BIS were also investigated. With regard to sex differences, the hypothesis predicts that females have higher levels of BIS than males. 

## 2. Materials and Methods

### 2.1. Participants

Ninety-three (40 males) athletes and non-athletes were recruited from three senior high schools and the Mid Sweden University. Of these, twenty-nine (15 males) participants were biathletes that had been admitted to a nationally approved sports program at a high school or sports academy aged M = 18.21, SD = 1.80 years, with biathlon experience of M = 7.62, SD = 3.03 years; thirty (12 males) were alpine skiers that had been admitted to a nationally approved sports program at a high school or sports academy aged M = 17.27, SD = 1.05 years, with alpine ski racing experience of M = 12.17, SD = 2.79 years; thirty-four (13 males) were non-athletes aged M = 17.50, SD = 1.29 years that had not been admitted to any form of sports program or sports academy. 

### 2.2. Measures

#### 2.2.1. Demographic Questionnaire 

Participants provided age, sex, and sport participation activity (e.g., sports played, times per week spent playing sports, and years spent actively playing sports), for descriptive data and grouping purposes. 

#### 2.2.2. The BIS/BAS Scales 

A Swedish version of the BIS/BAS scales [50] was used. The BIS/BAS scales have 24 items (4 of which are filler) that are rated on a 4-point Likert scale and comprise the 7-item Behavioral Inhibition System (BIS) scale and the 13-item Behavioral Approach System (BAS) scale. To differentiate FFFS and BIS in Carver and White’s BIS/BAS scales, Heym et al.’s [51] split was used. Consequently, in the current study, FFFS consists of 3 items (e.g., “Even if something bad is about to happen to me, I rarely experience fear or nervousness”), and BIS consists of 4 items (e.g., “Criticism or scolding hurts me quite a bit”). In the current sample, McDonald’s ω values for BAS-FS, BAS-DR, BAS-RR, and BAS total were 0.64, 0.79, 0.69, and 0.81, respectively. For BIS (4 items) and FFFS (3 items), ω values were 0.82 and 0.56, respectively. Due to its low value, the FFFS was not used in analyses.

#### 2.2.3. Stop-Signal Task

The stop-signal task was performed on a computer (Dell Latitude 7490, Round Rock, TX, USA) with a 14-inch screen. A gamepad (Logitech Gamepad F310, Lausanne, Switzerland) was used as an input device. Each trial started with the presentation of a white circular fixation ring (80 × 80 pixels) displayed at the center of a black background screen. After 500 ms, a white arrow was displayed, and on one-half of the trials, the arrow pointed to the right, and on the other half, the arrow pointed to the left. The direction of the arrow was randomized. The fixation ring and arrow remained on the screen for up to 1000 ms (limited hold), after which they disappeared, and the background screen was shown during an inter-stimulus interval. The inter-stimulus interval was randomized and ranged between 1000–2000 ms. For the go task, the participant responded as fast as possible with a left or right button press using the index finger of the right and left hand. For the stop task (25 percent of the trials), the participant attempted to stop his/her response when a stop signal was presented. The arrow changed color to red for 150 ms after an adjustable stop-signal delay (SSD) relative to the onset of the go stimulus, which indicated that the response should have been withheld. To dynamically adjust SSD, the tracking procedure [52] was used, and this was performed throughout the experiment. The initial SSD was set to 250 ms, and if inhibition was successful on a stop trial, SSD was increased by 50 ms on the following stop trial, and if not, the SSD was reduced by 50 ms on the following stop trial. 

### 2.3. Procedure

Participants were given a brief overview of this study before signing the informed consent. Prior to the stop-signal task, the participants were asked to complete a web-based questionnaire containing the demographic questions and the BIS/BAS scales. Participants were placed in a chair in front of the screen with a viewing distance of 0.5 m. The gamepad was held with both hands, with the index fingers placed on the right and left buttons in front of the gamepad. The participants were instructed to respond as quickly and accurately as possible by indicating with their index fingers whether the arrow pointed to the left or to the right and to inhibit their response if the arrow turned red. Successful inhibition was defined as no press of the trigger buttons. After the instructions, there was a block of 20 training trials. The training block was followed by three blocks of 128 trials, each block containing 96 go trials and 32 stop trials. The direction of the arrows and stop trials were randomized in sets of four. Between each block, there was a one-minute break, and the next block was started by the participant. The participants were instructed that stopping would not always be possible and that stopping and going was equally important. A low proportion of stop signals (25 percent) was applied to avoid strategies, such as slowing down the response to the go signal, with the aim to ensure successful inhibition [16]. 

### 2.4. Data Treatment

Accuracy was calculated for all go trials, and errors were commission errors (left–right mistakes) or omission errors (failing to press either button). For reaction time (goRT), the individual mean was calculated for all correct responses. For signal–respond (SRT), the individual mean was calculated for all responses on unsuccessful stop trials. The stop-signal reaction time (SSRT) was estimated from the distributions of go trial response time and the go stimulus–stop stimulus–onset asynchronies (the stop-signal delay, SSD) on stop trials. For SSD, a tracking procedure was applied, but that procedure did not generate a success rate of stopping at 50 percent. There are many different ways to estimate SSRT, and in the current study, the so-called “integration method” [53,54,55] was used. The main reason for that choice was that the integration method could be used when the success rate for stopping was less than 50 percent, and in addition, it has been suggested to be less sensitive to, for example, motivational strategies than other procedures. All RTs for go trials were rank-ordered, and the RT value at the percentile that corresponded to the percentage of failed inhibitions was determined on a per-subject basis (e.g., the RT at the 45th percentile of the go-RT distribution for a participant with 45 percent unsuccessful stop trials). The estimation of SSRT for each participant was performed by subtracting the average SSD from the RT value attained from the tracking procedure.

### 2.5. Statistical Analyses

Differences between groups were assessed with a one-way ANOVA. In order to obtain an overview of the associations between the variables of interest, Pearson’s correlation coefficient was computed to assess the strength and direction of the relationship between SSRT and the trait variable BIS, as well as for the other study variables. Independent samples *t*-tests were used to examine sex differences in SSRT, the trait variables BIS and BAS, and differences in years of experience between alpine skiers and biathletes. To test the independence of the race model, mixed-model ANOVAs were used. Partial eta-squared (ηp^2^), Cohen’s *d*, and correlation coefficients (*r*) were used to report effect sizes. For interpretation of the effect sizes, the following cutoffs were used: for ηp^2^: ≥0.01 and <0.09 small, ≥0.09 and <0.25 medium, and ≥0.25 large; for Cohen’s *d*: 0.2 = small, 0.5 = medium, and 0.8 = large; for r: 0.10 = small, 0.30 medium, and 0.50 large [56]. The significance level was set at α < 5%, and for all analyses, JASP was used.

## 3. Results

Table 1 shows descriptive statistics of the different parameters of the stop-signal task and the trait variables (BIS and BAS) for each group. Table 2 shows descriptive statistics of the different parameters of the stop-signal task and the trait variables (BIS and BAS) for males and females and the total sample.

BIS was significantly correlated with SSRT, with higher levels of BIS being associated with increased SSRT (see Table 3). There were also significant correlations between SSRT and Accuracy and GoRT and Accuracy, with increased GoRT and SSRT being associated with decreased Accuracy.

For SSRT, there was no significant main effect of group (alpine skiers, biathletes, and non-athletes) (*F*(2, 90) = 0.12, *p* = 0.89, ηp^2^ = 0.003). There was a significant difference in SSRT between males (M = 136.78, SD = 48.90) and females (M = 157.76, SD = 34.98), *t*(91) = −2.41, *p* = 0.018, *d* = 0.51, with a small to medium effect size. There was a significant difference in BIS scores between males (M = 10.23, SD = 2.57) and females (M = 13.34, SD = 2.15), *t*(91) = −6.40, *p* < 0.001, *d* = 1.36, with a large effect size. The difference in BAS scores between males (M = 40.38, SD = 5.39) and females (M = 41.19, SD = 4.81) was not significant (*t*(91) = −0.77, *p* = 0.45, *d* = 0.16). There was a significant difference in years of experience between alpine skiers (M = 12.17, SD = 3.03) and biathletes (M = 7.62, SD = 2.79; *t*(57) = −5.99, *p* < 0.001, *d* = 1.56), with a large effect size. The correlation between years of experience and SSRT in the entire group of athletes (n = 57) was not significant (*r* = −0.08).

### 3.1. Explorative Analysis

To examine if the difference between males and females in SSRT was influenced by levels of BIS, an analysis of covariance was conducted with BIS as a covariate. There was no significant effect of gender on SSRT after controlling for the effect of levels of BIS (*F*(1, 90) = 2.25; *p* = 0.14; ηp^2^ = 0.02).

### 3.2. Assumption Testing of the Race Model

A mixed-model [Trial type (GoRT vs. SRT, within-subject factor) × Group (non-athletes vs. biathletes vs. alpine skiers, between-subject factor)] showed a main effect of trial type indicating that GoRT was significantly slower than SRT (*F*(1, 90) = 143.50, *p* < 0.001, ηp^2^ = 0.62). The main effect of group was not significant (*F*(2, 90) = 1.24, *p* = 0.30, ηp^2^ = 0.03), and there was no interaction between trial type and group (*F*(2, 90) = 0.66, *p* = 0.52, ηp^2^ = 0.02). A mixed-model ANOVA [Trial type (GoRT vs. SRT, within-subject factor) × Sex (females vs. males, between-subject factor)] showed a main effect of trial type indicating that GoRT was significantly slower than SRT (*F*(1, 90) = 139.86, *p* < 0.001, ηp^2^ = 0.61). The main effect of group was not significant (*F*(1, 91) = 0.19, *p* = 0.66, ηp^2^ = 0.002). However, a significant interaction was found between trial type and sex, indicating that the differences in reaction time between go-trials and stop-trials in females were significantly larger than in males (*F*(1, 91) = 4.39, *p* = 0.04, ηp^2^ = 0.05). For descriptive statistics, see Table 2. 

## 4. Discussion

The results showed that contrary to the hypothesis, there were no significant differences in response inhibition (as indexed by SSRT) between biathletes, alpine skiers, and non-athletes. As the effect size approached null with respect to the variation in SSRT, it could be concluded that the effect in the current sample was not dependent on the type of sport nor based on being an athlete or non-athlete. Krenn et al. [26] suggested that differences in EFs could depend on the type of sport since different types of sports might challenge the EFs in different ways. Since several of the studies on response inhibition (e.g., [4,7,8,21,22]) used athletes from sports classified as strategic sports, it is possible that there are certain characteristics of the strategic sports that have been investigated, which may place higher cognitive demands, as suggested by Krenn et al. [26]. In the current study, the biathletes had fewer years of experience as active in their sport than alpine skiers. However, there was no significant correlation between the entire group of athletes and SSRT, which indicated that years of experience did not confound the results. Furthermore, the null findings suggest that it is possible that the two sports (included in the current study) involve skill executions that put demands on the EF inhibition that are different from the response inhibition, which takes place in a non-specific stop-signal task. It is also evident that, although biathlon and alpine skiing differ in terms of involvement of fine motor skills, it did not manifest as differences in response inhibition in the current sample. 

Although we did not find any differences between the groups, we found a significant difference in response inhibition between males and females, showing significantly shorter SSRT for males. However, the effect size was weak; only six percent of the variation in SSRT could be explained by sex. The evidence of sex differences in response inhibition is inconclusive (see [18]); nevertheless, although with a weak effect size observed in the present study, it is in line with the sex difference in SSRT that Gaillard et al. [19] found. Our data also show sex differences in the trait variable BIS, with females scoring significantly higher than males, which was in line with the hypothesis and supports previous findings [39,40,41,42]. In addition, we found a significant but weakly positive correlation between BIS scores and SSRT, but the explorative analysis in which BIS scores were controlled for in the comparison of SSRT between males and females showed that the effect of sex on SSRT was no longer significant. There were no differences between males and females in the BAS. Taken together, these results suggest that it is important to consider and further investigate how motivational dispositions, such as the BIS, interact with response inhibition. 

Behavioral data for both females and males in the current study indicated that the assumptions of the race model were not violated. That is, the GoRT was slower than SRT within each group, which allowed for a reliable estimation of SSRT. However, an important aspect to consider when interpreting the empirical findings within the field is that there is a great diversity in how EFs are operationalized and measured. Thus, response inhibition, as operationalized in the stop-signal paradigm, is one among other methods for the assessment of inhibitory control. Friedman and Miyake [11] point out that inhibition as a construct should not be regarded as a unifying mechanism, and Matzke et al. [17] emphasize that SSRT is a global concept that reflects more than a single inhibitory process. A consensus guide for stop-signal tasks [55] was recently published as a result of the large variation between studies using the paradigm, variations that rang from SST design to data treatment and analysis. Therefore, it is possible that the contradictory findings in studies on response inhibition in athletes and non-athletes using the stop-signal paradigm, to some extent, could be explained by large variations in, for example, SST design. In the current study, the stop-signal paradigm was chosen due to the fact that the SST allowed for a measurement of response inhibition and has been shown to initiate goal conflict [47,48], therefore enabling the possibility to explore the relationship of the BIS and response inhibition. 

This study has some limitations. Regarding the sampling of athletes and non-athletes and level of expertise, the athletes in the sample were, at best, at a sub-elite level with different levels of experience. In addition, other factors that are known for their association with inhibition, such as aerobic fitness, and other EFs, such as, for example, working memory or mental flexibility, were not included in the current study. With respect to the choice of SST design, the aim was to use a design that would allow for reliable estimation of SSRT in line with the recommendations provided by Verbruggen et al. [55] for group comparison. However, the consequence of that choice is the ecological validity in that it is not comparable to the demands that the athletes are exposed to in their specific sport.

## 5. Conclusions

The findings in the current study indicate that the interactions between sex, BIS, and SSRT need to be further explored since the understanding of these interactions could have meaningful implications in sports. More specifically, our results suggest that it might be meaningful to not only investigate differences in response inhibition in different types of sports or in athletes and non-athletes but the contribution of sex differences and motivational dispositions (such as the BIS) on response inhibition in conjunction with different types of sports also need to be considered and further explored.

## Figures and Tables

**Table 1:** Descriptive statistics of the study variables for the three groups.

Variable	Alpine Akiing (n = 30)		Biathlon (n = 29)		Non-Athletes (n = 34)	
	M	SD	M	SD	M	SD
GoRT (ms)	560.75	152.96	540.75	146.31	595.93	153.85
SRT (ms)	508.37	138.47	497.36	138.08	553.33	149.33
SSRT (ms)	145.63	40.93	150.47	39.16	149.99	47.67
SSD (ms)	394.09	145.56	376.43	135.84	428.03	141.35
*p* (respond│signal)	0.46	0.07	0.47	0.08	0.45	0.06
Accuracy (%)	93.25	9.29	95.00	7.20	92.29	7.41
BIS	12.10	3.25	11.62	2.44	12.24	2.65
BAS	42.37	4.82	40.34	4.54	39.91	5.50

Note. M = mean; SD = standard deviation; GoRT = reaction time go trials; SRT = signal–respond reaction time; SSRT = stop-signal reaction time; SSD = stop-signal delay; *p* (respond│signal) = probability of responding given a stop signal; BIS = Behavioral Inhibition System; BAS = Behavioral Approach System.

**Table 2:** Descriptive statistics of the study variables for males, females and the total sample.

Variable	Males (n = 40)		Females (n = 53)		Total (n = 93)	
	M	SD	M	SD	M	SD
GoRT (ms)	570.52	162.57	565.00	143.93	567.38	151.39
SRT (ms)	533.58	159.41	512.16	130.15	521.37	143.04
SSRT (ms)	136.78	48.90	157.76	34.98	148.73	42.60
SSD (ms)	416.96	153.74	388.94	131.78	400.99	141.53
*p* (respond│signal)	0.45	0.08	0.47	0.07	0.46	0.07
Accuracy (%)	92.37	10.23	94.28	6.56	93.50	8.00
BIS	10.23	2.54	13.34	2.15	12.00	2.78
BAS	40.38	5.39	41.19	4.81	40.84	5.06

Note. M = mean; SD = standard deviation; GoRT = reaction time go trials; SRT = signal–respond reaction time; SSRT = stop-signal reaction time; SSD = stop-signal delay; BIS = Behavioral Inhibition System; BAS = Behavioral Approach System.

**Table 3:** Correlations for study variables.

Variable	1	2	3	4	5
1 GoRT (ms)	–				
2 SSRT (ms)	0.23 *	–			
3 Accuracy (%)	−0.59 **	−0.22 *	–		
4 BIS	−0.06	0.21 *	0.08	–	
5 BAS	−0.16	−0.11	−0.00	0.06	–

Note. GoRT = reaction time go trials; SSRT = stop-signal reaction time; BIS = Behavioral Inhibition System; BAS = Behavioral Approach System; * *p* < 0.05, two-tailed. ** *p* < 0.01, two-tailed.

## Data Availability

The data that support the findings of this study are available upon request from the corresponding author.
